# Astaxanthin ameliorates inflammation, oxidative stress, and reproductive outcomes in endometriosis patients undergoing assisted reproduction: A randomized, triple-blind placebo-controlled clinical trial

**DOI:** 10.3389/fendo.2023.1144323

**Published:** 2023-03-20

**Authors:** Sahar Rostami, Ashraf Alyasin, Mojtaba Saedi, Saeid Nekoonam, Mahshad Khodarahmian, Ashraf Moeini, Fardin Amidi

**Affiliations:** ^1^ Department of Anatomy, School of Medicine, Tehran University of Medical Sciences, Tehran, Iran; ^2^ Department of Infertility, Shariati Hospital, Tehran University of Medical Sciences, Tehran, Iran; ^3^ Department of Obstetrics and Gynecology, Shariati Hospital, Tehran University of Medical Sciences, Tehran, Iran; ^4^ Department of Infertility, Arash Women’s Hospital, Tehran University of Medical Sciences, Tehran, Iran; ^5^ Department of Gynecology and Obstetrics, Arash Women’s Hospital, Tehran University of Medical Sciences, Tehran, Iran; ^6^ Department of Endocrinology and Female Infertility, Reproductive Biomedicine Research Center, Royan Institute for Reproductive Biomedicine, Academic Center for Education, Culture, and Research (ACECR), Tehran, Iran

**Keywords:** endometriosis, assisted reproductive techniques (ARTs), female infertility, astaxanthin (AST), antioxidants

## Abstract

**Purpose:**

In a randomized, triple-blind, placebo-controlled clinical trial (RCT) including 50 infertile women with endometriosis candidate for assisted reproductive techniques (ART), we studied the effect of Astaxanthin (AST) on pro-inflammatory cytokines, oxidative stress (OS) markers, and early pregnancy outcomes.

**Methods:**

Before and after 12 weeks of AST treatment (6 mg per day), blood serum and follicular fluid (FF) samples were collected from 50 infertile women with endometriosis stage III/IV undergoing ART. Pro-inflammatory cytokines (IL-1β, IL-6, and TNF-α) and OS markers (malondialdehyde [MDA], superoxide dismutase [SOD], catalase [CAT], and total antioxidant capacity [TAC]) were measured in the serum and FF. ART outcomes were also compared between the groups.

**Results:**

Increased serum levels of TAC (398.661 ± 57.686 *vs*. 364.746 ± 51.569; P = 0.004) and SOD (13.458 ± 7.276 *vs*. 9.040 ± 5.155; P = 0.010) were observed after AST therapy in the treatment group. Furthermore, serum MDA (14.619 ± 2.505 *vs*. 15.939 ± 1.512; P = 0.031) decreased significantly following antioxidant treatment. In addition, significantly lower serum levels of IL-1β (4.515 ± 0.907 *vs*. 6.8760 ± 0.8478; P = 0.000), IL-6 (5.516 ± 0.646 *vs*. 5.0543 ± 0.709; P = 0.024) and TNF-α (2.520 ± 0.525 *vs*. 2.968 ± 0.548; P = 0.038) were observed after AST treatment. In addition, AST supplementation led to an improved number of oocytes retrieved (14.60 ± 7.79 *vs*. 9.84 ± 6.44; P = 0.043), number of mature (MII) oocytes (10.48 ± 6.665 *vs*. 6.72 ± 4.3; P = 0.041), and high-quality embryos (4.52 ± 2.41 *vs*. 2.72 ± 2.40; P = 0.024).

**Conclusion:**

AST pretreatment can modulate inflammation and OS in endometriosis-induced infertile patients. ART outcomes also improved after 12 weeks of AST therapy. Our results suggest that AST can be a potential therapeutic target for infertile patients with endometriosis undergoing ART.

## Introduction

Endometriosis, the presence of endometrial-like tissue (glands and stroma) outside the uterine cavity, is a chronic estrogen-dependent inflammatory disease affecting approximately 10-15% of reproductive-age women and 35–50% of women with pelvic pain and/or infertility worldwide (1). There is an enigmatic association between endometriosis and infertility. Distorted pelvic anatomy, changes in the peritoneal fluid milieu, endocrine and ovulatory abnormalities, compromised ovarian function and impaired folliculogenesis, diminished ovarian reserve due to surgery, disrupted endometrial receptivity, and inability to have regular intercourse due to dyspareunia might all have detrimental effects on natural conception (2–4).

A complex network of molecular, cellular, and hormonal factors could help unravel the underlying pathogenic mechanism for declined reproductive outcomes of endometriosis patients (5). Endometriosis lesions are present in a unique microenvironment rich in inflammation, steroid hormones (especially estrogen), oxidative stress (OS), and iron originating from erythrocytes and menstrual debris in the peritoneal cavity (6, 7). Reduced activity of endogenous antioxidant enzymes and increased accumulation of reactive oxygen species (ROS) have been detected in endometriosis patients (8, 9). Elevated OS caused by the accumulation of iron in the peritoneal fluid and endometriotic cells contributes to the activation of the nuclear factor−κB (NF-κB) pathway in endometriotic lesions and peritoneal macrophages involved in the inflammatory reaction caused by endometriosis (10). NF-κB pathway activity leads to increased levels of pro-inflammatory cytokines, including interleukin (IL)-1β, IL-6, IL-8, tumor necrosis factor (TNF)-α, and interferon (IFN)-γ, in the peritoneal fluid, serum, and follicular fluid (FF) of women with endometriosis (11, 12). These factors contribute to poor oocyte quality, impaired fertilization, and embryo implantation failure (5, 13).

The assisted reproductive technique (ART) is the main treatment option for endometriosis-induced infertility (14). It may not, however, be absolutely and solely successful, as fertility rates are lower in patients with stage I/II endometriosis, and follicle-stimulating hormone (FSH) requirements are higher and fewer oocytes are collected in patients with stage III/IV endometriosis (13, 15). In recent decades, the use of complementary and alternative medicine in treating endometriosis has been welcomed due to its promising efficiency and few side effects (9, 16). Since redox dysregulation plays a vital role in the pathophysiology of endometriosis, a considerable number of studies have indicated the positive effects of antioxidants such as vitamin C, vitamin E, resveratrol, curcumin, melatonin, and epigallocatechin-3-gallate (EGCG) as a supplement along with common endometriosis treatments (17).

Astaxanthin (dihydroxy-4,4′-dione- β,β′-carotene: AST), called the king of antioxidants, is a red-orange, lipid-soluble xanthophyll photo-pigmented ketocarotenoid isolated from the alga *Haematococcus pluvialis* with an antioxidant activity 10 times stronger than that of other natural carotenoids (18). In addition to an outstanding singlet oxygen-quenching activity, this multi-target pharmacological agent has been shown to exert great immunomodulatory, anti-inflammatory, anti-proliferative, anti-apoptotic, anti-diabetic, and neuroprotective properties (19).

AST targets several molecules and pathways, including phosphoinositide 3-kinases (PI3K)/Akt and Janus kinase-2/signal transducer and activator of transcription-3 (JAK2/STAT3) signaling pathways, NF-κB family, mitogen-activated protein kinases (MAPKs), nuclear factor erythroid 2-related factor 2 (Nrf2)/heme oxygenase-1 (HO-1), and peroxisome proliferator-activated receptor gamma (PPARγ) to efficiently modulate the dynamic oxidant: antioxidant equilibrium (19). Various preclinical studies have shown that AST exerts antioxidant effects mainly *via* the Nrf2/HO-1 antioxidant signaling pathway, and increases the expression of its antioxidant target genes, including the phase II biotransformation enzyme (20). Moreover, AST alleviates oxidative damage to DNA by lowering 8-hydroxy-20 deoxyguanosine (8-OHdG), inhibits lipid oxidation by decreasing malondialdehyde (MDA), a decomposition product of peroxidized polyunsaturated fatty acids in membrane lipids, and reduces 8-isoprostane (ISP), an eicosanoid product of the free radical-catalyzed oxidation of primarily arachidonic acid. It can also increase plasma total antioxidant capability (TAC) and enhance the activity of antioxidant enzymes, such as superoxide dismutase (SOD), glutathione peroxidase (GSH-Px), and paraoxonase (PON) (21).

Furthermore, AST suppresses the expression of scavenger receptors, controlled by NF−κB, the main mediator of the inflammatory response through inhibiting IκB-α degeneration and the NF-κB nuclear translocation (22). As a result, the expression of downstream pro-inflammatory genes, including IL−1β, IL-6, TNF-α, chemokines, cyclooxygenase−2 (COX−2), inducible nitric oxide synthase (iNOS), and matrix metalloproteinases (MMPs) in macrophages and other cell types are downregulated by AST (20). Furthermore, AST causes a decrease in cytokines *via* inhibition of the MAPK signaling pathway by reducing the induction and expression of extracellular-signal-regulated kinase (ERK1/2), c-Jun N-terminal kinases (JNK), and p38 MAP Kinase (19, 23).

Fertility-enhancing properties of AST have been established in various *in vitro* and *in vivo* studies. In previous experimental studies (24–27), the protective role of AST against intracellular ROS levels has been demonstrated in mice and human granulosa cells and oocytes by up-regulating the phase II enzymes caused by Nrf2/HO-1 activation. In a previous clinical trial (28), the positive effects of AST supplementation were reported on OS and ART outcomes in infertile women with polycystic ovary syndrome (PCOS). However, no previous study has investigated the effect of AST on infertile endometriosis patients. We conducted a randomized placebo-controlled clinical trial (RCT) to study the effect of AST supplementation on OS, inflammation, and reproductive outcomes in endometriosis patients undergoing ART.

## Materials and methods

### Patient enrollment and study design

This was a prospective, parallel, randomized, triple-blind, placebo-controlled clinical trial (RCT) with a 1:1 ratio. We enrolled 50 infertile endometriosis patients presenting for ART at Omid Fertility Clinic, Tehran, Iran, between December 2021 and September 2022. Endometriosis patients classified as moderate (stage III) or severe (stage IV) according to the American Society of Reproductive Medicine criteria, 1997 (29) who met the inclusion criteria were randomly assigned to AST (n = 25) and placebo groups (n = 25). Eligible trial participants included patients 20 to 40 years old suffering from infertility caused by stage III/IV endometriosis confirmed by video laparoscopy and histopathological tests, with 18.5 < BMI < 30 kg/m^2^, regular menstrual cycles, and no history of surgical treatments. Pregnant women, breast-feeders, or women using hormones or IUDs in the last 3 months prior to the first intervention, and those taking antioxidant drug treatments were not included. Patients with concomitant male factor infertility (especially non-obstructive azoospermia) or gynecological diseases leading to infertility, such as PCOS and tubal factor were excluded prior to randomization. Patients with a history of autoimmune disorders, abnormalities in the uterine cavity, such as fibroids and polyps, pelvic inflammatory disease, cancer, diabetes, chronic infectious diseases, or any other immune-affecting exposure were also excluded. Participants who became pregnant or those who started using hormonal drugs during the intervention were also excluded from the trial. Patients’ demographic and clinical data were documented, including age, BMI, type of infertility (primary or secondary), and duration of infertility. Their follicular phase hormonal profile (serum levels of anti-Müllerian hormone [AMH], follicle-stimulating hormone [FSH], luteinizing hormone [LH], estradiol [E2], and progesterone [P]), ovarian stimulation parameters and ART outcomes were also recorded and analyzed.

### Treatment randomization and blinding

**Figure 1:**
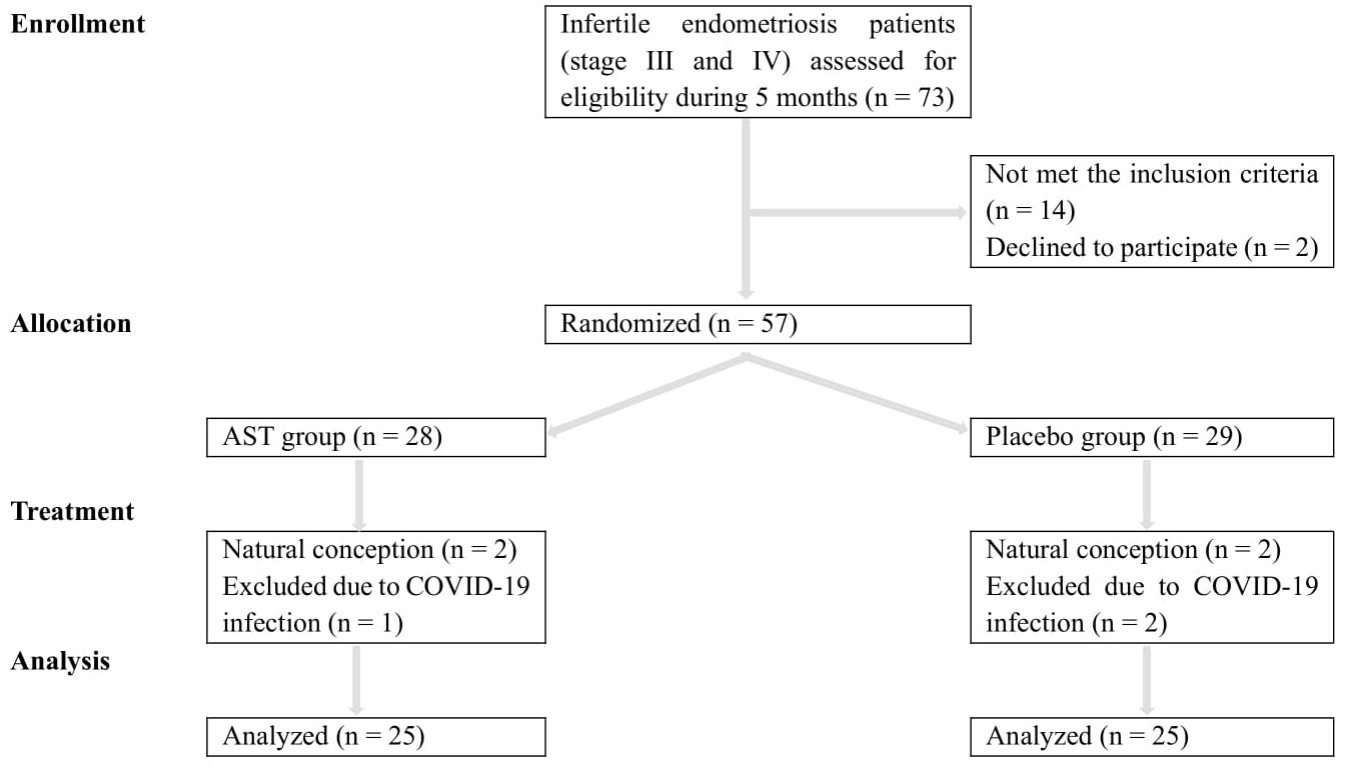
Patient enrolment and CONSORT diagram.

Eligible patients were randomly assigned to the AST or placebo groups (1:1) using the balanced block randomization method within a block size of 4 by an independent statistician. This trial was a triple-blind study. All patients, researchers, statisticians, embryologists, and laboratory personnel were blinded to the individual treatment allocation. The Consolidated Standards of Reporting Trials (CONSORT) diagram (**Figure 1**) shows the distribution of participants through the trial.

### Trial procedures

All infertile endometriosis patients candidate for ICSI protocol who visited Omid Fertility Clinic and met the inclusion criteria were recruited. Before and alongside the routine gonadotropin-releasing hormone (GnRH) antagonist ovarian stimulation protocol, 6 mg daily of oral AST or placebo capsules (AstaReal^®^ astaxanthin; AstaReal Co., Ltd., Tokyo, Japan) were administered to the treatment and placebo groups, respectively for 12 weeks (from Day 1 of two menstrual cycles before starting controlled ovarian stimulation [COS] until oocyte pick-up [OPU]). Placebo capsules (produced by the same company) were identical to AST capsules in color, size, shape, packaging, and taste. The dose and duration of the intervention were determined according to the previous studies, which showed that AST in a daily dose of 2 to 24 mg exhibits antioxidant properties with no safety concerns and side effects after at least a 3-week intervention (30–32). Weekly phone calls and monthly visits were used to check on the proper use of medications and their possible side effects. Patients were instructed to maintain their usual lifestyle and to refrain from taking nutritional supplements except for prenatal folic acid (Apovital^®^, Folsäure 800, Germany; 800 μg/day). All subjects were asked to return any remaining capsules on the day of the OPU to assess the adherence rate.

### Blood and FF collection and biochemical measurements

Sample collections were carried out following the guidelines suggested by the World Endometriosis Research Foundation (33). Blood samples (10 ml venous blood) were obtained from all patients before and after the intervention (on the day of OPU) to evaluate OS markers (the serum levels of TAC, MDA, SOD, and CAT activities) and pro-inflammatory cytokines (IL-1β, IL-6, and TNF-α). The blood samples were centrifuged immediately (1500 rpm for 10 min, 4°C), and serum was separated and stored in aliquots at − 80°C until further assessment. FF was aspirated only from the first follicle during the OPU to minimize blood contamination. The samples were centrifuged (3000 rpm for 10 min), and the clarified supernatants were collected. The FF aliquots were also promptly stored at − 80°C until further analysis.

OS markers and cytokines were measured in duplicate, in a blinded manner, in pairs (pre/post-intervention) simultaneously, and in the same analytical run to reduce systematic error and inter-assay variations. Measurements of serum and FF levels of OS markers were performed using the human enzyme-linked immunosorbent assay (ELISA) kits (Zellbio, GmbH, Germany), according to the manufacturer’s instructions. The kit sensitivity was 0.5 U/mL for CAT, 1 U/mL for SOD, 0.1 µM for MDA, and 0.1 mM for TAC detection. A microplate reader (BioTek, Winooski, USA) was used to measure absorbance at wavelengths specific to each marker. Pro-inflammatory cytokine concentrations were determined in serum and FF by high sensitivity human ELISA system using commercially available human kits (Karmania Pars Gene company; KPG, Iran), following the manufacturer’s instruction. The kit sensitivity was 2 pg/mL, and the absorbance was measured at 420 nm *via* a microplate reader (BioTek, Winooski, USA).

### COS protocol

The GnRH antagonistic protocol combined with the whole embryo freezing strategy is more efficient for ovulation induction in the Iranian endometriosis population. Thus, patients were submitted to a flexible antagonist protocol. Recombinant follicle-stimulating hormone (rFSH) (150–300 IU/day, Gonal-F^®^, Merck Serono SA, Switzerland) was administered at the beginning of the cycle and proceeded until the human chorionic gonadotropin (hCG) trigger for pituitary down-regulation. Serial transvaginal sonography (TVS) was conducted to evaluate the ovarian response. The GnRH (gonadotropin-releasing hormone) antagonist, Cetrorelix acetate (Cetrotide 0.25 mg, Merck Serono SA, Switzerland), was administered when 2 or more follicles reached ≥ 14 mm in diameter. Cetrotide was discontinued when at least 2 follicles achieved a diameter of ≥ 18 mm, and 10,000 IU hCG (Ovitrelle, Merck Serono SA, Switzerland) was administered for the ultimate oocyte maturation. The OPU was performed under an ultrasound guide 36 h ( ± 2 h) following the trigger. The participants were all subjected to the standard intracytoplasmic sperm injection (ICSI) protocol. In line with local clinical practice, to prevent ovarian hyperstimulation syndrome (OHSS) and as a strategy to improve clinical outcomes, all embryos were cryopreserved on Day 3/5, and 2 or 3 embryos were transferred 2 cycles later. Single embryo transfer (SET) was performed if 2 embryos were unavailable.

### ART outcomes

Two hours following OPU, hyaluronidase enzyme (Sigma^®^, USA) was used to denude cumulus-oocyte complexes (COCs). The retrieved oocytes were assessed for quality and maturity under a stereo microscope (Olympus SZX7, Tokyo, Japan) and were classified as germinal vesicle (GV), metaphase I (MI), and metaphase II (MII). ICSI was performed on injectable MII oocytes, and fertilization [the presence of 2 pronuclei (2 PN) and 2 polar bodies (2 PBs)] was assessed 16-18 hours following ICSI.

Reproductive outcomes, including the number of oocytes retrieved, the number of MII oocytes, oocyte maturity rate (the number of normal MII oocytes per the total number of normal oocytes retrieved, expressed as a percentage (34)), fertilization rate (the number of oocytes with 2PN/2PB, 16-18 h post-insemination ×100 per the number of MII oocytes injected (35)), high-quality embryos (grade A and B cleavage embryos based on the ASEBIR criteria (36)), chemical pregnancy rate (the number of pregnancies with a positive serum β-hCG test 14 days after ET per the number of ET cycles, expressed as a percentage (37)), clinical pregnancy rate (the number of pregnancies with a definitive clinical sign in ultrasound (gestational sac, heart rate, etc.) 5-6 weeks after ET per the number of ET cycles, expressed as a percentage (38)), and multiple pregnancy rate (the number of multiple pregnancies ×100 per the number of clinical pregnancies) were also collected.

### Sample size and statistical analysis

The serum level of TAC was the primary outcome. According to Choi et al.’s study (39) and a 15% loss-to-follow-up, a sample size of 50 (25 in each study group) would yield 80% power and a significance level of 0.05 to detect a difference in the mean serum TAC. We tested the data for normality using the Kolmogorov–Smirnov test. AST and placebo groups were compared using an independent sample t-test. Student’s paired t-test was used to compare pre- and post-intervention markers in each group. Mann–Whitney U test was used to investigate data without normal distribution. Pearson’s correlation was used to evaluate the correlations between the parameters. Chemical, clinical, and multiple pregnancy rates were compared using Fisher’s exact and Pearson’s chi-squared tests. The correlations between the serum and FF levels of the OS markers and cytokines with the ART outcomes were investigated using Pearson’s correlation coefficient. All statistical analyses were performed in SPSS (SAS Institute Inc, version 22, Cary, NC, USA). Data were presented as mean ± standard deviation (SD). A P value of less than 0.05 was considered significant.

### Ethical approval

The trial was approved by the Deputy of the Research and Ethics Committee of TUMS (approval date: 2021, December 19; code: IR.TUMS.MEDICINE.REC.1400.1085) and conducted following the Declaration of Helsinki, the International Council for Harmonisation Guidelines for Good Clinical Practice and applicable regulatory requirements. The study protocol was also registered in the Iranian Registry of Clinical Trials (registration code: IRCT20220625055274N1). Here we present a part of the RCT registered in the IRCT. All participants signed written informed consent to use the capsules and provide serum and discarded follicular fluid (FF) samples collected during OPU for research purposes. Before obtaining informed consent, the researchers explained the antioxidant properties, effects, and consumption instructions.

## Results

### Baseline characteristics

As depicted in the CONSORT flowchart, a total of 57 patients were randomized and exposed, of whom 50 (25 in each group) were enrolled and treated from December 2021 to May 2022 (**Figure 1**). Demographic and clinical data are presented in **Table 1**. There were no statistically significant differences in age (33.33 ± 4.97 vs. 32.08 ± 5.09; P = 0.242), BMI (23.17 ± 1.82 vs. 24.31 ± 2.60; P = 0.961), and duration of infertility (3.56 ± 3.19 vs. 2.96 ± 1.54; P = 0.102), AMH (2.72 ± 2.02 vs. 2.40 ± 1.50; P = 0.368), FSH (3.20 ± 2.27 vs. 4.56 ± 2.79; P = 0.371), LH (5.19 ± 3.60 vs. 8.86 ± 10.21; P = 0.777), estradiol (93.22 ± 68.16 vs. 88.21 ± 45.26; P = 0.776), and progesterone (2.84 ± 5.77 vs. 2.22 ± 3.75; P = 0.302) at the onset of the study between the two groups. Most patients in both groups (76% of the AST group (n = 19) and 64% of the placebo group (n = 16)) were diagnosed with primary infertility. All 50 patients completed the study. According to pill count-back on returned bottles, adherence rates were 91% and 94% in the AST and placebo groups, respectively. The patients reported no adverse effects or toxicity during the intervention.

**Table 1:** Demographic characteristics, clinical data, and endocrine profile of the participants.

	AST (n = 25)	Placebo (n = 25)	P-value
**Age (years)**	33.33±4.97	32.08±5.09	0.242
**BMI (kg/m^2^)**	23.17±1.82	24.31±2.60	0.961
**Duration of infertility (years**)	3.56±3.19	2.96±1.54	0.102
**AMH (µg/mL)**	2.72±2.02	2.40±1.50	0.368
**FSH (mIU/mL)**	3.20±2.27	4.56±2.79	0.371
**LH (mIU/mL)**	5.19±3.60	8.86±10.21	0.777
**E2 (pg/mL)**	93.22±68.16	88.21±45.26	0.776
**P (ng/mL)**	2.84±5.77	2.22±3.75	0.302

AST, Astaxanthin group; BMI, Body Mass Index [weight (kg)/height (m2)]; AMH, Anti-müllerian Hormone; FSH, Follicle-stimulating Hormone; LH, Luteinizing Hormone; E2, Estradiol; P, Progesterone. Data are presented as mean ± SD. A P-value less than 0.05 is considered significant.

### Serum and FF antioxidants and OS profile

Pre- and post-intervention concentrations of OS markers and cytokine levels results are shown in **Table 2**. There were no statistically significant differences in the serum and FF levels of the markers between the two groups before AST supplementation. Comparing baseline values, no statistically significant difference was seen in the serum or FF levels of the markers in the placebo group after treatment. Increased serum levels of TAC (398.661 ± 57.686 vs. 364.746 ± 51.569; P = 0.004) and SOD (13.458 ± 7.276 vs. 9.040 ± 5.155; P = 0.010) were observed after AST therapy in the treatment group compared to the baseline values. Serum MDA (14.619 ± 2.505 vs. 15.939 ± 1.512; P = 0.031) also decreased significantly following antioxidant treatment. At the same time, the concentration of CAT remained unchanged after AST administration (11.338 ± 4.778 vs. 13.420 ± 9.535; P = 0.304). No significant differences were seen in TAC (P=0.118), MDA (P = 0.662), SOD (P = 0.908), or CAT (P = 0.993) levels in the FF between AST and placebo groups.

**Table 2:** OS markers and cytokine levels in two groups.

	AST, pre-intervention serum levels	AST, post-intervention serum levels	Paired P-value	Placebo, pre-intervention serum levels	Placebo, post-intervention serum levels	Paired P-value	P-value between groups	AST, FF levels	Placebo, FF levels	t-test P-value
**MDA**	15.939821±1.512291	14.619420±2.505294	0.031^*^	19.568685±3.464909	21.392248±4.035089	0.059	0.000^****^	0.132324±0.030408	0.136272±0.28587	0.662
**SOD**	9.040516±5.155055	13.458493±7.276019	0.010^*^	9.391183±5.007821	9.077471±4.587139	0.817	0.032^*^	9.820102±5.462483	9.660697±4.204471	0.908
**CAT**	13.420228±9.535983	11.338269±4.778701	0.304	10.830017±8.545939	13.272669±8.355258	0.279	0.321	0.648546±0.082670	0.648880±0.171007	0.993
**TAC**	364.746301±51.569541	398.661250±57.686710	0.004^***^	393.730496±55.267005	406.175507±64.271907	0.341	0.666	314.443782±73.355726	287.858695±38.991069	0.118
**IL-1β**	6.8760±0.8478	4.5152±0.9078	0.000^****^	6.8045±1.6090	6.4580±0.9847	0.326	0.000^****^	4.2228±0.8870	4.2410±1.1094	0.324
**IL-6**	5.5165±0.6466	5.0543±0.7099	0.042^*^	5.1905±0.7789	5.5208±0.5218	0.063	0.98	4.0231±1.3622	4.8346±2.1064	0.113
**TNF-α**	2.9688±0.5487	2.5200±0.5255	0.038^*^	2.8750±0.6267	3.0102±0.7495	0.367	0.062	2.7352±0.0645	3.0887±0.7425	0.016^*^

AST, Astaxanthin group; MDA, malondialdehyde; SOD, superoxide dismutase; CAT, catalase; TAC, total antioxidant capacity; IL-1β, interleukin 1β; IL-6, interleukin 6; TNF-α, tumor necrosis factor-α. Data are presented as mean ± SD. A P-value less than 0.05 is considered significant (*P < 0.05, ***P < 0.001, and ****P < 0.0001).

### Serum and FF cytokine parameters

Among cytokine parameters, significantly lower serum levels of IL-1β (4.515 ± 0.907 vs. 6.8760 ± 0.8478; P = 0.000) and TNF-α (2.520 ± 0.525 vs. 2.968 ± 0.548; P = 0.038) were observed after AST treatment (**Table 2**). However, no significant effect was observed on serum IL-6 concentrations (5.5165 ± 0.6466 vs. 5.0543 ± 0.7099; P = 0.042). There were no significant differences in the FF IL-1β levels between the AST and the placebo group after the intervention (all P > 0.05). Nevertheless, FF IL-6 (P = 0.024) and TNF-α (P = 0.016) were significantly lower in the AST group than in the placebo group.

### Comparison of ovarian stimulation parameters and ART outcomes

Comparing ovarian stimulation parameters and ART outcomes, the number of oocytes retrieved (14.60 ± 7.79 vs. 9.84 ± 6.44; P = 0.043), the number of MII oocytes (10.48 ± 6.665 vs. 6.72 ± 4.3; P = 0.041), and high-quality embryos (4.52 ± 2.41 vs. 2.72 ± 2.40; P = 0.024) improved significantly after AST therapy. The number of transferred embryos was similar in the two groups (2.24 ± 0.43 vs. 2.04 ± 0.73; P = 0.203). The fertilization rate (P = 0.382) and multiple pregnancy rate (P = 0.741) were also similar (**Table 3**). No significant difference was detected in chemical (14/25 (56%) vs. 11/25 (44%); two-tailed P = 0.414), clinical (11/25 (44%) vs. 10/25 (40%); two-tailed P = 0.241), or multiple pregnancy rates (4/25 (16%) vs. 3/25 (12%); two-tailed P = 1.000) between the two groups.

**Table 3:** ART outcomes between AST and placebo groups.

	AST (n = 25)	Placebo (n = 25)	P-value
**Number of oocytes**	14.60±7.79	9.84±6.44	0.043^*^
**GV**	1.36±1.52	1.20±1.29	0.692
**MI**	1.08±1.28	1.20±1.25	0.664
**MII**	10.48±6.665	6.72±4.32	0.041^*^
**Oocyte maturity rate (MII %)**	75.52±13.54	64.64±20.40	0.055
**Fertilized oocytes**	8.48±5.76	5.40±3.90	0.059
**Fertilization rate (%)**	79.64±16.67	73.61±28.07	0.382
**Number of frozen embryos**	7.52±4.98	4.88±3.45	0.060
**High-quality embryos**	4.52±2.41	2.72±2.40	0.024^*^
**Frozen embryos**	7.52±4.98	4.88±3.45	0.060
**Number of transferred embryos**	2.24±0.436	2.04±0.735	0.203

AST, Astaxanthin group; Oocyte maturity rate, number of MII oocytes/ number of oocytes retrieved × 100; Fertilization rate, number of fertilized oocytes/ numbers of injected MII oocytes × 100; high-quality embryos, grade A/B cleavage embryos; Fertilization rate, number of injected oocytes with 2 pronuclei (2 PN) and 2 polar bodies (2 PBs) 16-18 h post-insemination/ number of MII oocytes injected ×100. Data are presented as mean ± SD. A P-value less than 0.05 is considered significant (*P < 0.05).

### Correlation analysis

Finally, as shown in **Tables 4** and **5**, TAC, MDA, SOD, and CAT concentrations in serum and FF were analyzed for correlations. Correlation analysis showed that serum IL-1β correlated negatively with fertilization rate (r_s_ = -0.416; P = 0.039). Serum TNF-α also correlated negatively with MII rate (r_s_ = -0.680; P = 0.000), fertilization rate (r_s_ = -0.446; P = 0.025), and high-quality embryos (r_s_=-0.582; P = 0.002). There were positive correlations between FF MDA and fertilization rate (r_s_ = 0.420; P = =0.036) and high-quality embryos (r_s_ = 0.449; P = 0.025). There were strong positive correlations between FF CAT and MII rate (r_s_ = 0.744; P = 0.000), fertilization rate (r_s_ = 0.843; P = 0.000), and high-quality embryos (r_s_ = 0.679; P = 0.000). FF TAC and total oocytes retrieved (r_s_ = 0.587; P = 0.002), MII rate (r_s_ = 0.812; P = 0.000), fertilization rate (r_s_ = 0.979; P=0.000), and high-quality embryos (r_s_ = 0.976; P = 0.000) correlated positively as well. Surprisingly, we found a positive correlation between FF IL-6 and total oocytes retrieved (r_s_ = 0.414; P = 0.040). Eventually, FF TNF-α and MII rate (r_s_ = -0.723; P = 0.000), fertilization rate (r_s_ = -0.818; P = 0.000) and high-quality embryos (r_s_ = -0.683; P = 0.000) correlated strongly and negatively.

**Table 4:** Correlations between OS markers and ART outcomes.

	Total oocytes		MII rate		Fertilization rate		Number of frozen embryos		High-quality embryos		Chemical pregnancies		Clinical pregnancies	
	r_s_	P	r_s_	P	r_s_	P	r_s_	P	r_s_	P	r_s_	P	r_s_	P
**Serum MDA**	-0.85	0.686	-0.254	0.220	-0.380	0.061	0.021	0.921	-0.357	0.080	-0.253	0.222	-0.217	0.296
**Serum SOD**	0.056	0.792	0.339	0.098	0.245	0.238	0.041	0.846	0.245	0.238	-0.121	0.564	-0.133	0.527
**Serum CAT**	0.006	0.978	-0.077	0.716	0.161	0.441	0.029	0.891	0.164	0.435	-0.082	0.697	-0.108	0.608
**Serum TAC**	0.345	0.091	0.305	0.138	0.317	0.122	0.387	0.056	0.323	0.116	0.165	0.430	0.048	0.819
**FF MDA**	0.115	0.585	0.278	0.179	0.420	0.036	0.052	0.803	0.449	0.025	-0.224	0.282	-0.386	0.056
**FF SOD**	0.258	0.213	0.181	0.358	0.239	0.249	0.101	0.630	0.243	0.242	-0.107	0.612	-0.267	0.197
**FF CAT**	0.291	0.158	0.744	0.000	0.843	0.000	-0.213	0.306	0.679	0.000	-0.173	0.408	-0.145	0.489
**FF TAC**	0.587	0.002	0.812	0.000	0.979	0.000	-0.105	0.618	0.976	0.000	-0.047	0.823	-.0185	0.375

MDA, malondialdehyde; SOD, superoxide dismutase; CAT, catalase; TAC, total antioxidant capacity; MII rate (oocyte maturity rate), number of MII oocytes/ number of oocytes retrieved × 100; Fertilization rate, number of fertilized oocytes/ numbers of injected MII oocytes × 100; High-quality embryos, grade A/B cleavage embryos; Chemical pregnancies, pregnancies with a positive serum β-hCG; Clinical pregnancies, pregnancies with a definitive clinical sign. r_s_, Pearson correlation coefficient.

**Table 5:** Correlations between pro-inflammatory cytokines and ART outcomes.

	Total oocytes		MII rate		Fertilization rate		Number of frozen embryos		High-quality embryos		Chemical pregnancies		Clinical pregnancies	
	r_s_	P	r_s_	P	r_s_	P	r_s_	P	r_s_	P	r_s_	P	r_s_	P
**Serum IL-1β**	-0.147	0.483	-0.093	0.658	-0.416	0.039	0.181	0.387	-0.356	0.081	-0.193	0.355	-0.011	0.960
**Serum IL-6**	-0.125	0.551	0.000	0.999	0.027	0.900	-0.46	0.826	-0.007	0.975	0.029	0.890	0.069	0.745
**Serum TNF-α**	-0.0219	0.294	-0.680	0.000	-0.446	0.025	-0.263	0.204	-0.582	0.002	0.260	0.210	0.210	0.314
**FF IL-1β**	0.171	0.413	0.121	0.564	-0.234	0.261	0.124	0.554	-0.204	0.329	-0.197	0.345	-0.087	0.679
**FF IL-6**	0.414	0.040	0.241	0.246	0.251	0.226	0.284	0.170	0.339	0.097	0.225	0.280	0.202	0.332
**FF TNF-α**	-0.308	0.134	-0.723	0.000	-0.818	0.000	0.127	0.545	-0.683	0.000	0.086	0.684	-0.009	0.965

IL-1β, interleukin 1β; IL-6, interleukin IL-6; TNF-α, tumor necrosis factor-α. MII rate (oocyte maturity rate), number of MII oocytes/ number of oocytes retrieved × 100; Fertilization rate, number of fertilized oocytes/ numbers of injected MII oocytes × 100; High-quality embryos, grade A/B cleavage embryos; Chemical pregnancies, pregnancies with a positive serum β-hCG; Clinical pregnancies, pregnancies with a definitive clinical sign. r_s_, Pearson correlation coefficient.

## Discussion

Since OS and inflammation are interrelated and involved in the pathophysiology of endometriosis-associated infertility, in a randomized, triple-blind, placebo-controlled clinical trial on 50 infertile endometriosis patients candidate for ART, we investigated the antioxidative, anti-inflammatory, and fertility-enhancing effects of AST supplementation. We indicated that a 12-week supplementation with 6 mg/day of AST could effectively alleviate OS and inflammation and enhance ART outcomes.

This trial showed that women using AST for 12 weeks achieved significantly higher serum levels of TAC and SOD. Serum MDA was also decreased significantly following antioxidant treatment. At the same time, the concentration of CAT remained unchanged after AST administration. However, no significant changes were detected in the CAT levels. This is consistent with published RCTs performed in Korea (39, 40), which aimed to assess the effect of supplementary AST on lipid profiles and OS in overweight and obese subjects and showed a significantly lower MDA and ISP but a remarkably higher TAC after a 12-week AST supplementation. Lower MDA levels after 45 days of AST supplementation were also reported by Baralic et al. (41). They investigated the effect of AST on the OS status of young soccer players. A previous RCT (28) was conducted to investigate the effect of a 40-day AST administration on OS markers in PCOS patients and showed a significant increase in the serum TAC and CAT levels in the intervention group. No differences were observed in MDA and SOD serum levels.

A statistically significant decrease was observed in the FF TNF-α and IL-6 levels between the AST and the placebo group. However, dietary AST did not affect OS markers in the FF of endometriosis patients. Although AST supplementation led to a higher TAC level, it was not statistically significant. A previous study on PCOS patients undergoing ART (28) also showed no improvement in the FF levels of OS markers, except CAT, after AST supplementation. This is also in line with Gong et al. (42) who reported no significant changes in the FF TAC, MDA, and SOD levels in the FF of PCOS patients treated with growth hormone. However, the total oxidant status (TOS) and the OS Index (OSI) of the FF reduced. Thus, further studies are warranted to measure TOS, OSI, total antioxidant status (TAS), pro-oxidant-antioxidant balance (PAB), and other biomarkers of OS (8-OHdG, GSH-Px, PON, etc.) after antioxidant therapy. Moreover, in future studies, the presence of AST and its oxidation fragments should be analyzed in the FF.

Significantly lower serum levels of IL-1β and TNF-α, but no considerable changes in serum IL-6, were observed following the intervention. In an RCT by Park et al. (2010) (43) on healthy women, plasma IFN-ɣ and IL-6, but not TNF, increased after 8 weeks of AST supplementation. An increase in the circulating total T and B cells was observed as a result of enhanced humoral immune responses, including IFN-ɣ and IL-6 production. Cai et al. (2019) (44) also showed that AST treatment could attenuate inflammatory factors (TNF-α and IL-6) significantly by inhibiting MAPK/NF-κB signaling pathway in a mouse model of lipopolysaccharide (LPS)-induced sepsis and acute lung injury *in vitro* and *in vivo*. According to Wan et al. (45), treating bovine endometrial epithelial cell line with AST could alleviate LPS-induced production of IL-6 and TNF- α, improve the cellular activity of SOD and CAT, and decrease apoptosis.

Endometrial explants potentially expose the developing follicle, the oocyte, sperm, and embryos to high levels of ROS generated by inflammation. The damage to gametes and embryos can be prevented by antioxidant supplementation (15). Although limited by sample size, in this study the number of oocytes, mature oocytes, and high-quality embryos improved significantly after AST therapy. Thus, AST could promisingly protect oocytes and embryos from oxidative damage. Fertilization rate, not significantly different between the two groups, is informative of gamete quality and maturity and reflects operator competence. Moreover, although we excluded male factor patients, fertilization may be influenced by sperm quality. We indicated no significant intergroup difference in the number of fertilized oocytes on Day 1. However, the fertilized oocytes in the AST group contributed to a higher number of good-quality (grade A and B) embryos. Thus, AST may enhance the function of oocytes and improve their capability to produce higher-quality embryos. However, no significant difference was detected in ET outcomes, chemical and clinical pregnancies, between the two groups. These results are compatible with our previous clinical trial on PCOS patients (28). In addition to our small sample size, various potential variables affect pregnancy outcomes, such as male gamete status, embryologist and clinician skills in ET, potential effects of cryopreservation in frozen ET cycles, and endometrial receptivity (38). Further studies with larger sample size comparing outcomes in fresh and frozen cycles may be required.

Correlation analysis showed that serum IL-1β correlated negatively with fertilization rate. Nevertheless, increased IL-1β concentrations have been previously reported to be beneficial as high IL-1β in patients presenting male or unexplained infertility correlated with higher fertilization, implantation, and pregnancy rates (46, 47). However, they concluded that the positive association should be confirmed in patients who present different obstetric disorders leading to infertility, including endometriosis. Serum TNF-α also correlated negatively with MII rate, fertilization rate, and high-quality embryos. Promising results of treating endometriosis-associated infertility with TNF-α blockers supports our findings (48). There were positive correlations between FF MDA and fertilization rate and high-quality embryos. This finding is in line with Liu et al. (49), who showed a significant association between FF MDA and embryo quality indicators in PCOS patients. Prieto et al. (50) also found a positive correlation between levels of FF MDA and the implantation rate. There were strong positive correlations between FF CAT and MII rate, fertilization rate, and high-quality embryos. FF TAC and total oocytes retrieved, MII rate, fertilization rate, and high-quality embryos correlated positively as well. In Jana et al.’s study (51), decreased TAC in FF correlated with poor oocyte and embryo quality and low fertilization rate. Pasqualotto et al. (52) also found a positive correlation between FF TAC and pregnancy outcomes. We found a positive correlation between FF IL-6 and total oocytes retrieved. Bedaiwy et al. (53) also reported a significantly higher FF IL-6 in pregnant cycles. However, no correlation was shown between FF IL-6 and oocyte yield, embryo parameters, and fertilization rate according in a study by Altun et al. (54). Since they observed a negative correlation between FF IL-6 levels with increased chance of clinical pregnancy, they suggested endometrial receptivity as the main target for adverse effects of elevated IL-6 levels. Eventually, FF TNF-α and MII rate, fertilization rate, and high-quality embryos correlated strongly and negatively. This is in line with Lee et al. (55), who found higher FF TNF-α concentrations in poor-quality oocytes.

To the best of our knowledge, this is the first randomized, triple-blind, placebo-controlled clinical trial evaluating the effect of AST supplementation on OS markers, inflammatory cytokines, and ART outcomes in infertile endometriosis patients. Furthermore, a homogenous study population from the whole country undergoing uniform treatment practices was included. It is important to note, however, that our study had some limitations. Our study only includes patients undergoing assisted reproduction, so the results may not be generalizable to the entire endometriosis patient population. A larger sample size is required to analyze pregnancy outcomes. Besides, time constraints prevented us from evaluating late pregnancy outcomes, including live birth rate, the most relevant standard of success in ART. In addition, our analysis was limited to frozen ICSI cycles without assessing pregnancy outcomes in fresh cycles.

## Conclusion

In conclusion, AST administration could be a promising supplement to combat the oxidative stress and inflammatory reaction associated with endometriosis. This pharmacological agent may also enhance oocyte and embryo quality in endometriosis patients presenting to ART clinics.

## Data availability statement

The original contributions presented in the study are included in the article/supplementary material. Further inquiries can be directed to the corresponding author.

## Ethics statement

The trial was approved by the Deputy of the Research and Ethics Committee of TUMS (approval date: 2021, December 19; code: IR.TUMS.MEDICINE.REC.1400.1085). The patients/participants provided their written informed consent to participate in this study.

## Author contributions

FA and AA designed the experiments and contributed reagents/materials/analysis tools. SR collected the data and performed the experiments. SN performed the statistical analysis, critically appraised the data, and reviewed the manuscript. SR and MS wrote the first draft of the manuscript. MK and AM contributed to manuscript revision and editing. All authors contributed to the article and approved the submitted version.
